# Controversies and Future Directions in Management of Acute Appendicitis: An Updated Comprehensive Review

**DOI:** 10.3390/jcm13113034

**Published:** 2024-05-22

**Authors:** Dushyant Singh Dahiya, Hamzah Akram, Aman Goyal, Abdul Moiz Khan, Syeda Shahnoor, Khawaja M. Hassan, Manesh Kumar Gangwani, Hassam Ali, Bhanu Siva Mohan Pinnam, Saqr Alsakarneh, Andrew Canakis, Abu Baker Sheikh, Saurabh Chandan, Amir Humza Sohail

**Affiliations:** 1 Division of Gastroenterology, Hepatology & Motility, The University of Kansas School of Medicine, Kansas City, KS 66160, USA; 2 Department of Internal Medicine, Hamilton Health Sciences, Hamilton, ON L8N 3Z5, Canada; 3 Department of Internal Medicine, Seth GS Medical College and KEM Hospital, Mumbai 400012, India; 4 Department of Internal Medicine, Ayub Medical College, Abbottabad 22020, Pakistan; 5 Department of Internal Medicine, Dow University of Health Sciences, Karachi 74200, Pakistan; 6 Department of Internal Medicine, King Edward Medical University, Lahore 54000, Pakistan; 7 Department of Gastroenterology and Hepatology, University of Arkansas for Medical Sciences, Little Rock, AR 72205, USA; 8 Division of Gastroenterology, Hepatology and Nutrition, East Carolina University/Brody School of Medicine, Greenville, NC 27858, USA; 9 Department of Internal Medicine, John H. Stroger, Jr. Hospital of Cook County, Chicago, IL 60612, USA; 10 Department of Internal Medicine, University of Missouri-Kansas City, Kansas City, MO 64110, USA; 11 Division of Gastroenterology and Hepatology, University of Maryland School of Medicine, Baltimore, MD 21201, USA; 12 Department of Internal Medicine, University of New Mexico, Albuquerque, NM 87131, USA; 13 Division of Gastroenterology and Hepatology, Creighton University School of Medicine, Omaha, NE 68178, USA; 14 Department of Surgery, University of New Mexico, Albuquerque, NM 87131, USA

**Keywords:** appendicitis, appendectomy, interval appendectomy, endoscopy

## Abstract

Globally, acute appendicitis has an estimated lifetime risk of 7–8%. However, there are numerous controversies surrounding the management of acute appendicitis, and the best treatment approach depends on patient characteristics. Non-operative management (NOM), which involves the utilization of antibiotics and aggressive intravenous hydration, and surgical appendectomy are valid treatment options for healthy adults. NOM is also ideal for poor surgical candidates. Another important consideration is the timing of surgery, i.e., the role of interval appendectomy (IA) and the possibility of delaying surgery for a few hours on index admission. IA refers to surgical removal of the appendix 8–12 weeks after the initial diagnosis of appendicitis. It is ideal in patients with a contained appendiceal perforation on initial presentation, wherein an initial nonoperative approach is preferred. Furthermore, IA can help distinguish malignant and non-malignant causes of acute appendicitis, while reducing the risk of recurrence. On the contrary, a decision to delay appendectomy for a few hours on index admission should be made based on the patients’ baseline health status and severity of appendicitis. Post-operatively, surgical drain placement may help reduce postoperative complications; however, it carries an increased risk of drain occlusion, fistula formation, and paralytic ileus. Furthermore, one of the most critical aspects of appendectomy is the closure of the appendiceal stump, which can be achieved with the help of endoclips, sutures, staples, and endoloops. In this review, we discuss different aspects of management of acute appendicitis, current controversies in management, and the potential role of endoscopic appendectomy as a future treatment option.

## 1. Introduction

Acute appendicitis ranks among the most common surgical emergencies globally, with an estimated lifetime incidence of 7–8% [1]. The highest incidence of appendicitis is seen in the second and third decades of life [2]. The commonly accepted pathophysiology implicated in the development of appendicitis is the blockage of the appendiceal lumen, resulting in edema, vascular congestion, localized ischemia, and, in severe instances, appendiceal rupture, potentially leading to widespread peritonitis and sepsis [3,4]. In the United States (US), the incidence of perforated appendicitis is estimated to be approximately 29 cases per 100,000 person years [5]. Owing to the considerable life-threatening complications associated with appendicitis, appendectomy is one of the most performed surgical procedures in modern medicine. Interestingly, the incidence of appendicitis has gradually trended down over the years for reasons that mostly remain unclear [6,7]. For over a century, open appendectomy was the standard of care for appendicitis. However, in recent decades, surgical advancement has led to laparoscopic appendectomy becoming the preferred intervention due to lower postprocedural infection rates, shorter length of hospital stay and recovery, minimal pain, and improved patient satisfaction [8,9].

Acute appendicitis is primarily classified into uncomplicated and complicated appendicitis. Uncomplicated appendicitis refers to an inflamed but intact appendix. Complicated appendicitis is an umbrella term encompassing perforated appendicitis, often with bacterial peritonitis, abscess, and phlegmon or fistula formation, among other complications. [10]. Strategies for optimal management are specifically tailored to the nature, etiology, and severity of appendicitis. Historically, for uncomplicated non-perforated appendicitis, the standard of care has been appendectomy owing to fear that untreated appendicitis leads to eventual perforation, thereby drastically increasing mortality risk [9]. However, in recent years, the choice of treatment is dependent on several factors, including the presence of perforation or abscess, age, comorbidity profile, frailty, immune status, associated surgical risks, and the potential for appendiceal malignancy, among others.

In the current literature, there are numerous knowledge gaps that exist in the management of acute appendicitis. These include decisions on management (surgical versus non-surgical), optimal timing of appendectomy, indications and timing of interval appendectomy (IA), pre-operative medicine, type of surgical intervention in high-risk populations (open versus laparoscopic), intra-operative techniques, post-operative antibiotics, and the need for drain placement [11]. Our updated review aims to address current treatment approach and knowledge gaps associated with the management of appendicitis, providing an in-depth evidence-informed analysis of the myriad of intra- and perioperative treatment modalities as well as the strategies currently in use and their appropriate indications. Furthermore, we also discuss emerging therapeutic modalities that may ultimately decrease morbidity and mortality associated with appendicitis.

## 2. Methods

A detailed systematic search was performed through February 2024 in Ovid EBM Reviews, ClinicalTrials.gov, Ovid Embase (1974+), Ovid Medline (1946+ including epub ahead of print, in-process, and other non-indexed citations), Scopus (1970+), and Web of Science (1975+). The literature search included studies published in all languages. For non-English studies, a language translation service was used to convert them into English. Furthermore, conference abstracts and bibliography from all articles were also reviewed for additional studies. We utilized numerous combinations of keywords in our literature search such as: ‘appendicitis’, ‘management’, ‘interval appendectomy’, ‘knowledge gaps’, ‘antibiotics’, ‘surgery’, ‘stump closure’, ‘pre-operative’, ‘post-operative’, ‘costs’, ‘timing’, ‘drain’, ‘laparoscopic appendectomy’, and ‘open appendectomy’. Three authors (DSD, HA, and AG) performed the literature search and reviewed all citations individually. Two authors (SC and AHS) reviewed all studies individually to check if the included studies reported on pertinent data. All studies were included irrespective of whether they were performed in an inpatient or outpatient setting, prospective or retrospective fashion, had short or longer follow-up time, and country of origin, as long as they provided appropriate data. In cases of multiple studies from a single group of authors or patient subset, data from the largest or most recent comprehensive study was included.

## 3. Management of Acute Appendicitis

The optimum management strategy for acute appendicitis varies depending on the patient cohort, with different modalities being employed to manage appendicitis in adults, children, immunocompromised individuals, and pregnant women. Generally, among healthy adults, both non-operative management (NOM) and surgical appendectomy are viable therapeutic choices [12]. In children, uncomplicated appendicitis has traditionally been treated with appendectomy; however, there is increasing evidence for NOM among healthy children with no risk factors and favorable radiographic findings [13,14]. In pregnant women, management with antibiotic therapy alone is not recommended due to an increased risk of failure, a significant risk of fetal loss with perforation, and a lack of sufficient safety data on this approach [15,16,17]. Among elderly and immunocompromised patients, diminished immune response may result in delayed diagnosis of appendicitis, thereby increasing the risk of perforation [18]. Hence, NOM is not considered a viable approach, and prompt surgical intervention is the ideal choice. Timing of appendectomy (emergent versus interval) and effective antibiotic regimens are other key considerations during management.

## 4. Role of Non-Operative Management in Acute Appendicitis

NOM involves appropriate effective antibiotics and aggressive fluid hydration for patients to prevent the need for surgical intervention. In this approach, appendectomy is only considered for individuals with poor clinical response to conservative management, continued deterioration on antibiotics, or those experiencing recurrent appendicitis. Moreover, NOM is ideal for patients who are deemed poor surgical candidates. However, patients opting for this management strategy must be counseled on the increased potential risks of disease progression despite antibiotic treatment, appendicitis recurrence, or, in rare instances, missed underlying neoplasms [12]. Furthermore, NOM is contraindicated in immunocompromised and pregnant patients, as well as patients with hemodynamic instability or a history of inflammatory bowel disease, due to paucity of high-quality data, especially randomized evidence, to support the use of this management strategy.

Effective antibiotic therapy, either intravenous or oral, forms the crux of NOM for acute appendicitis. Data suggest that an overwhelming majority of patients (90%) treated with antibiotics successfully avoid surgery. However, the remaining patients who fail to respond to antibiotics alone warrant surgical intervention. Nonetheless, accurately predicting an individual’s responses to NOM is challenging [19]. Favorable response is monitored by trends in clinical parameters, such as leukocytosis and symptoms of appendicitis, e.g., pain, nausea, and vomiting [20,21]. Furthermore, the study by Erikkson et al. demonstrated that patients treated with NOM have lower pain level and analgesic requirements compared to those treated surgically [22]. However, there are some data demonstrating that surgical intervention may be superior to NOM. In a recent meta-analysis conducted by Zagales et al., which included 12 clinical trials with 3703 patients of acute appendicitis, surgical appendectomy had a significantly higher effectiveness than NOM (98.4% vs. 73.3%, *p* < 0.0001), but the authors did not find a statistical difference on the length of hospital stay between the two groups [23]. Similarly, a meta-analysis by Podda et al. demonstrated a higher treatment efficacy (based on a 1-year follow-up) in the surgical appendectomy cohort compared to the NOM group (98.3% vs. 75.9%, *p* < 0.0001) [24]. The authors also observed that the rate of complicated appendicitis with concurrent peritonitis, identified at the time of surgery in cases of recurrence, was higher in the NOM group compared to the surgically treated group (19.9% vs. 8.5%, *p* = 0.02). This study also demonstrated no significant difference in the length of hospital stay between the two groups [24]. Additionally, a few trials evaluating long-term outcomes have also reported a higher risk of recurrence (15–40%) of appendicitis when patients are managed nonoperatively [25,26]. Hence, further large-scale randomized controlled trials comparing the two therapeutic modalities, with carefully selected cohort of patients, is required to determine the ideal management strategy and embark change in current guidelines. From a patient perspective, owing to the novel nature of NOM in managing acute appendicitis, shared decision-making between the clinician and the patient is a prerequisite [27].

## 5. Role of Interval Appendectomy

Surgical removal of the appendix 8–12 weeks after the initial presentation of acute appendicitis is known as delayed or IA. It is often performed in cases of contained appendiceal perforation on initial presentation wherein an a nonoperative approach is preferred treatment modality [28]. Some experts recommend always performing an IA after the initial NOM to rule out neoplasm as the underlying etiology of acute appendicitis [29].

In a study by Carpenter et al., 28% of patients who underwent IA had an underlying neoplasm compared to only 1% in the immediate or index appendectomy group (*p* < 0.0001) [30]. Another study by Wright et al. supported these findings, with 12% of patients undergoing IA having neoplasms compared to only 0.5% of patients undergoing index appendectomy [31]. In another trial, the rate of incidental neoplasm discovery was as high as 29% among patients aged > 40 years [32]. Furthermore, performing an IA offers the advantage of reduced risk of recurrence of appendicitis [25,26]. However, this does not justify performing an IA because the rate of recurrence of symptoms after the successful NMO of perforated appendicitis is sufficiently low in asymptomatic patients [33,34].

## 6. Impact of Delayed Appendectomy on Index Admission

In the 21st century, our understanding of acute appendicitis evolved significantly from the late 19th century, as pioneered by Fitz et al., with a growing emphasis on early diagnosis and immediate surgical intervention [35]. This has led to a drastic reduction in the mortality rate associated with appendicitis from 50% to 15% [36]. However, recent decades have seen crucial advances in appropriate antibiotic quality and diagnostic radiology, enabling successful delayed appendectomies through accurate assessment of disease severity and infection control [37,38].

In management of acute appendicitis, controversy still exists regarding the necessity of immediate surgical intervention versus the feasibility of safely delaying the procedure until an opportune time. The landmark DELAY trial investigated the practice of delaying appendectomy for acute appendicitis versus immediate appendectomy [39]. Of the 127 randomized participants, one group underwent immediate surgery (*n* = 68), while the delayed group (*n* = 59) had surgery postponed for an average duration of 11 h [39]. The comparison of outcomes revealed no significant differences between the two groups [39]. Essentially, the authors demonstrated the non-inferiority of postponing surgery until the following morning compared to immediate surgical intervention [39]. Building on this premise, other retrospective studies also showed no statistical difference between the early and late surgery groups in terms of hospital length of stay, operative time, complication rate, perioperative morbidity, or 30-day readmission rate [40,41,42,43]. A meta-analysis of 45 studies, which included 152,314 patients, demonstrated that delaying appendectomy for up to 24 h is safe for patients without preoperative signs of complicated appendicitis [44]. On the other hand, Ditillo et al. observed that delayed appendectomy for acute appendicitis may result in increased severity of disease pathology, potentially leading to increased adverse outcomes [45]. Consequently, it is important to consider the nature and extent of the disease pathology and severity when making important treatment decisions. Therefore, patient baseline health status and appendicitis severity should be key considerations in the complex decision-making process.

The abovementioned studies underscore the critical importance of early accurate diagnosis of appendicitis and assessing its pathology before making clinical decisions. Furthermore, they highlighted the potential of high-quality Computer Tomography (CT) imaging to predict the safety and feasibility of delaying appendectomy [46]. To conclude, appendectomy must be performed early after the initial resuscitation in patients with acute appendicitis.

From a healthcare burden perspective, it is worth noting that delayed appendectomy can result in increased costs. A study revealed that the cost escalated to USD 9893 ± USD 497 in the subgroup wherein surgery was delayed for 12 h compared to USD 7766 ± USD 886 in the subgroup where the delay in surgical intervention was limited to 6 h [47].

## 7. Role of Perioperative Antibiotics

Preoperative antibiotics are guideline-recommended for patients with acute appendicitis [48]. This practice aims to reduce the occurrence of postoperative surgical site infections and intra-abdominal abscesses, both of which are well-documented complications of appendectomy [49]. Provision of pre-incisional antibiotics is a double-pronged strategy that aims to reduce bacterial load within the inflamed appendix, thus minimizing contamination risk and providing coverage for any contamination that may occur. This prophylactic approach is crucial in patients with complicated appendicitis. Additionally, patients with uncomplicated acute appendicitis also typically receive a preoperative dose of broad-spectrum antibiotics [50]. The World Social of Emergency Surgery guidelines recommend the administration of a single dose of broad-spectrum antibiotics within 60 min of the surgical incision [51]. This has demonstrated effectiveness in reducing wound infections and postoperative intra-abdominal abscesses, with no significant difference in the nature of the removed appendix [51].

The choice of antibiotics and appropriate coverage of the bacterial spectrum is crucial. Numerous antibiotic regimens have been investigated in this context. Studies have demonstrated the superior effectiveness of certain antibiotic combinations such as cefotaxime and metronidazole in reducing wound infections compared to other regimens such as metronidazole and ciprofloxacin [52].

## 8. Role of Postoperative Antibiotics

In appendectomies for complicated appendicitis, postoperative antibiotics are indicated to minimize the risk of infectious complications. However, the data on the use of postoperative antibiotics in patients with nonperforated appendicitis are less favorable. Studies suggest that a single preoperative antibiotic dose is sufficient to prevent postoperative complications in patients undergoing laparoscopic appendectomy for uncomplicated appendicitis, and postoperative antibiotics did not provide additional clinical benefit in this cohort, but, rather, may increase postoperative morbidity and length of hospital stay [48,53,54,55,56,57]. If perforation occurs, postoperative antibiotics are recommended and should be administered for a minimum period of 3–5 days until improvement in clinical and laboratory parameters is observed [49,55].

## 9. Role of Drain Placement

Surgical removal of the appendix can lead to a myriad of postoperative complications including wound infections (about 20%) and intra-abdominal abscesses (9–20%) [58]. Several approaches can be employed to prevent these postoperative complications, including insertion of intra-abdominal drains, delayed wound closure, and opting for laparoscopy instead of open surgery whenever possible [59].

Traditional teaching methods aim to prevent the collection of inflammatory debris, infection, blood, pus, and other fluids at the surgical site and aid in draining pre-existing collections. Through this mechanism, abdominal drains considerably lower bacterial burden and the resultant risk of surgical site infections [60]. In clinical practice, some surgeons routinely use prophylactic surgical drains during appendectomy for complicated appendicitis to minimize postoperative complications. Surgical drains are also a valuable tool in uncomplicated appendicitis as well because they reduce postoperative collections and abscess formation, typically at a rate of 1–2% [61].

In general, the use of abdominal drains is based on personal experience rather than strict evidence-based guidelines, as exemplified by the surgical truism, “When in doubt, drain,” first coined by Tait in 1905 [62]. However, use of surgical drains has several disadvantages such as drain blockage, potential hindrance in the healing process, prolonged hospital stay, and increased healthcare expenses [63,64]. Complications such as erosion into the abdominal viscera, fistula formation, and drain-associated complications, i.e., entrapment, displacement, kinking, or migration, are well documented [65]. A comprehensive meta-analysis by Petrowsky et al. demonstrated that in complicated appendectomies, surgical drains do not reduce postoperative complications and may, in fact, increase the risk of enterocutaneous fistulas (4.2–7.5%) and wound infections (43–85%) [66]. Drain placement can also contribute to intestinal obstruction and paralytic ileus. This obstruction can result from the drain’s mechanical presence (foreign object) or through the potential introduction of extra-abdominal bacteria, and thus, infection and scarring [67]. Furthermore, Cheng et al. observed that routine intra-abdominal drain placement after open emergency appendectomy for complicated appendicitis was not associated with reduced risk of intraperitoneal abscess formation [68]. In another systematic review and meta-analysis of 17 studies consisting of 4255 patients with complicated appendicitis, 1580 underwent abdominal drainage, while 2657 did not [69]. The authors did not find a significant difference in the abdominal collections between the two groups [69]. However, the no-drain group had a lower incidence of surgical site infection, fistulae, intestinal obstruction, and paralytic ileus [69].

In conclusion, although abdominal drains are commonly used after appendectomy, they are associated with serious adverse events. Furthermore, recent data have cast doubt on its efficacy in preventing postoperative complications. Surgical decisions should be tailored to individual circumstances, keeping in mind the latest evidence to ensure high-quality care.

## 10. Techniques for Closure of Appendiceal Stump

The most critical step in an appendectomy is appendiceal stump closure, which aims to prevent intra-abdominal complications resulting from leakage of fecal matter into the abdominal cavity. Numerous techniques have been utilized and investigated for this purpose, including endoclips, endoloops, staplers, and sutures [70]. The superiority of one technique over another has not yet been evaluated, and is an area of active research. Data comparing mechanical and ligation techniques showed no significant differences in intra- or postoperative complications between the two groups [71]. However, the costs of these techniques vary significantly. Hem-o-lok clips are the most cost-effective treatment option as they are about 11.9, 12.55, and 66.9 times less expensive than Vicryl, PDS loops, and Echelon staplers, respectively, for each laparoscopic appendectomy procedure [72]. For reference, a single Hem-o-lok clip has been priced at USD 2.49 [73].

Each of these techniques has specific characteristics and applications, as detailed below.

### 10.1. Endoclips

Endoclips are surgical tools that are placed endoscopically to secure the appendix stump. These may be metallic or biodegradable [74]. Their notable advantage is the ease of application leading to a reduced operative time and operative cost [75,76]. However, a major drawback of endoclips is their limited width and capacity. Although they have been used for appendiceal base diameters of up to 16 mm, their effectiveness declines for diameters over 1 cm and raises concern for a stump leak if applied for larger diameters [77,78]. In recent years, polymeric endoclips, known as Hem-o-lok clips, have emerged as an efficient and cost-effective modality for uncomplicated appendectomies [79]. They can be applied safely and significantly reduce laparoscopic procedure times and overall cost [73]. Furthermore, compared to Endoloops, Hem-o-lok has been shown to have notable advantages, including shorter surgical procedure times, reduced hospital length of stay, and lower cost, making it a potentially preferable modality for securing the appendiceal base [44,45]. Furthermore, data also show that Hem-o-lok clips are non-inferior to staplers across numerous clinical outcomes for both complicated and uncomplicated appendicitis [79].

### 10.2. Endoloops

Endoloops are surgical devices made of looped sutures which when tightened around the target tissue, seal it off by strangulation. Evidence on the use of endoloops demonstrates favorable outcomes, emphasizing their efficiency and low intraoperative complication risk [80]. Hence, they are a viable treatment option for surgeons. A major advantage of endoloops is their low cost, which results in significant financial advantages for the healthcare systems across the US considering how commonly appendectomies are performed [77,80]. Notably, some surgeons have developed handmade endoloops, further reducing costs and demonstrating the adaptability of this technique to various healthcare settings, especially in resource-poor settings [81]. However, it is crucial to recognize that proficiency in the use of the endoloop technique requires surgical expertise. A surgeon must have the procedural skills to place the knot and resect the appendix with minimal to no fecal contamination, highlighting the importance of excellent surgical training and experience [82].

### 10.3. Staplers

Staplers are sophisticated medical devices that, upon firing, form a closed staple line by clamping and then incising the intervening tissue, preventing contamination and associated complications. In appendectomy, staplers are particularly useful in complicated cases of severe inflammation [83]. Furthermore, their ability to seal a wide appendix base is a key notable advantage [84]. Additionally, staplers are user-friendly and do not require extensive training to place compared to other closure modalities [85]. However, their major disadvantage is the high cost, with a single endoscopic staple closure costing USD 545.60 [72,86].

### 10.4. Sutures

In laparoscopic appendectomy, the appendiceal base can be sutured. This involves either intra- or extracorporeal knot tying. Intracorporeal knot-tying demands superior surgical expertise compared to alternative management techniques and has a considerable learning curve [82]. Suture ligation of the appendix base is one of the most cost-effective techniques [87]. However, a major disadvantage is the fact that it is time-consuming and increases the operative time. While the previous literature has shown that suturing has a comparable safety profile to other techniques, recent systematic reviews have suggested otherwise, as they note higher complication risks associated with its use [87]. Given the high morbidity and mortality associated with stump leak, we suggest that this method be avoided during appendectomy.

## 11. Conversion of Laparoscopic into Open Appendectomy

Over the years, due to advancements in technology and minimally invasive surgical techniques, laparoscopic appendectomy has become the standard approach for acute appendicitis. However, about 5–10% of patients who undergo laparoscopic appendectomy may require conversion to an open procedure to improve patient outcomes [88]. Risk factors implicated in conversion from a laparoscopic to open appendectomy include patient age (>65 years old), male gender, complicated appendicitis (abscess formation, perforation), presence of adhesions, technical difficulty of the laparoscopic procedure, and total durations of symptoms [88]. Conversion of the procedure not only increases total operating time, but also healthcare utilization and procedural costs, while removing the beneficial attributes of laparoscopic surgery. Hence, in these high-risk patients, careful and personalized patient section is key in improving overall clinical outcomes and the safety of the procedure.

## 12. Future Perspectives and Directions

Despite recent advancements in the management of appendicitis, there is considerable room for improvement. Substantial efforts have been directed towards refining laparoscopic techniques; however, numerous issues remain unaddressed. For example, laparoscopy involves small abdominal wall incisions which help reduce the risk of postoperative surgical site infections, but do eliminate it entirely [89]. Cosmetics is also a major consideration in a certain subgroup of patients. Furthermore, limitations of laparoscopy itself include a two-dimensional visual field resulting in limited depth perception, and the need for specialized equipment that raises cost and access issues in low-resource healthcare settings [90].

A novel endoscopic approach has been explored to address challenges associated with conventional surgical techniques [90]. Endoscopic appendectomy is a noninvasive endoscopic technique that involves a complex series of meticulous steps, including marking the lesion border, performing a near-circumferential full-thickness resection around the lesion, dissecting the mesoappendix and appendicular artery via endoscopic access, and using snare-assisted traction for appendix dissection [91,92]. The procedure concludes with the closure of the defect with a double endoscopic suture technique after thorough cleansing of the area [93].

Endoscopic appendectomy offers numerous advantages over traditional surgical techniques. First, it allows the endoscopist to directly assess the extent of the appendiceal orifice lesions, enabling maximum preservation of the ileocecal valve and the surrounding colon. Second, endoscopic appendectomy provides more direct access to the appendiceal orifice lesion(s) and the appendix itself, minimizing the risk of injury to the surrounding structures. This is particularly relevant in patients with prior abdominal surgery and resultant adhesions. Third, endoscopic appendectomy is cosmetically superior because it leaves no surgical scars, thus avoiding incision-associated complications such as incisional hernias and wound infections [92]. Finally, endoscopic appendectomy ensures complete resection of the appendix and associated lesions, thus minimizing the risk of recurrent appendicitis. Although still its preliminary stages and not widely adopted due to the lack of guidelines on procedural techniques and patient selection, endoscopic appendectomy has been reported to be highly effective and safe with demonstrated complete resection in all cases without any major post-intervention complications [94].

To conclude, endoscopic appendectomy with its notable advantages of cost reduction, lower complication rates and, and favorable cosmesis compared to laparoscopic appendectomy is a major landmark in the management of appendicitis. Although it holds promise to become a ‘go to’ intervention in the future, additional research, refinement of technique, and specialized training is vital to ensure widespread adoption.

## 13. Conclusions

Acute appendicitis is a common surgical emergency worldwide, and appendectomy is one of the most frequently performed procedures. In cases of uncomplicated appendicitis, healthy adults and children with no significant risk factors may benefit from either NOM or surgery, as both are viable options. NOM is also the preferred choice for patients who are poor surgical candidates due to their comorbidities and functional status. However, in elderly, pregnant, or immunocompromised patients, a surgical approach is often preferred. IA is an area of controversy in the management of acute appendicitis. Although many patients who undergo IA may experience reduced recurrence rates, this does not necessarily justify performing IA as the likelihood of recurrence after successful NOM of appendicitis is already low in asymptomatic patients. Furthermore, the decision to delay appendectomy for a few hours after initial hospital admission should depend on the severity of appendicitis and the patient’s baseline health status. Prophylactic and postoperative antibiotics, along with the placement of a surgical drain, can help reduce postoperative complications in patients undergoing appendectomy. Ensuring proper closure of the appendiceal stump during surgical management is also a crucial step to prevent postoperative complications. Endoscopic appendectomy, a minimally invasive approach, has had a great impact on current management of acute appendicitis due to its lower complication rates and favorable cosmetic outcomes compared to laparoscopic and traditional surgical techniques. Additional research on efficacy and safety, refinement of endoscopic techniques, widespread specialized training, and innovations in equipment are still needed for complete adoption of endoscopic appendectomy as the primary treatment modality for acute appendicitis.
